# Longitudinal changes in EEG power, sleep cycles and behaviour in a tau model of neurodegeneration

**DOI:** 10.1186/s13195-020-00651-0

**Published:** 2020-07-15

**Authors:** C. M. Holton, N. Hanley, E. Shanks, P. Oxley, A. McCarthy, B. J. Eastwood, T. K. Murray, A. Nickerson, K. A. Wafford

**Affiliations:** Eli Lilly and Company, Erl Wood Manor, Windlesham, Surrey, GU20 6PH UK

**Keywords:** Alzheimer’s disease, Frontotemporal dementia, Behaviour, Sleep, Spectral power, Cognition, Neurodegeneration, Pathology, Tau, rTg4510

## Abstract

**Background:**

Disturbed sleep is associated with cognitive decline in neurodegenerative diseases such as Alzheimer’s disease (AD) and frontotemporal dementia (FTD). The progressive sequence of how neurodegeneration affects aspects of sleep architecture in conjunction with behavioural changes is not well understood.

**Methods:**

We investigated changes in sleep architecture, spectral power and circadian rhythmicity in the tet-off rTg4510 mouse overexpressing human P301L tau within the same subjects over time. Doxycycline-induced transgene-suppressed rTg4510 mice, tTa carriers and wild-type mice were used as comparators. Spectral power and sleep stages were measured from within the home cage environment using EEG electrodes. In addition, locomotor activity and performance during a T-maze task were measured.

**Results:**

Spectral power in the delta and theta bands showed a time-dependent decrease in rTg4510 mice compared to all other groups. After the initial changes in spectral power, wake during the dark period increased whereas NREM and number of REM sleep bouts decreased in rTg4510 compared to wild-type mice. Home cage locomotor activity in the dark phase significantly increased in rTg4510 compared to wild-type mice by 40 weeks of age. Peak-to-peak circadian rhythm amplitude and performance in the T-maze was impaired throughout the experiment independent of time. At 46 weeks, rTG4510 mice had significant degeneration in the hippocampus and cortex whereas doxycycline-treated rTG4510 mice were protected. Pathology significantly correlated with sleep and EEG outcomes, in addition to locomotor and cognitive measures.

**Conclusions:**

We show that reduced EEG spectral power precedes reductions in sleep and home cage locomotor activity in a mouse model of tauopathy. The data shows increasing mutant tau changes sleep architecture, EEG properties, behaviour and cognition, which suggest tau-related effects on sleep architecture in patients with neurodegenerative diseases.

## Introduction

Alzheimer’s disease (AD) and other forms of dementia such as frontotemporal dementia (FTD) are characterised by progressive neurodegeneration resulting in progressive loss of cognitive function and memory [1, 2]. The symptoms most affecting quality of life of dementia patients and their caregivers are neuropsychiatric symptoms, weight loss and sleep disturbances [3–5]. It is crucial to recognise and treat sleep problems in dementia patients since these are associated with cognitive and functional decline [6, 7] and worsening behavioural or psychological symptoms [8]. Both AD and FTD often present with desynchronisation of circadian rhythms, lower sleep efficiency, lower percentage of non-rapid-eye-movement (NREM) sleep and a greater frequency of arousals and awakenings [9–13]. These changes have recently been shown to correlate with pathological deficits produced my misfolded proteins such as Aβ and tau [14, 15]. A reduced percentage of REM or the deeper stages of NREM sleep are the most consistently reported in patients with mild to moderate AD. All sleep disturbances worsen with increasing severity of the disease [6, 16]. The consistent pattern of sleep impairment and EEG spectral power observed in AD warrant in-depth investigation in longitudinal studies.

Cognitive decline in AD correlates with degeneration of the hippocampus, medial temporal and medial parietal lobes [17–20]. Many different rodent mouse models have been generated to mimic some of these properties, and some have been shown to exhibit changes in sleep and EEG at particular time points [15, 21–23]. To model aspects of tau-related neurodegeneration and cognitive decline, the repressible rTg(tet-o-Tau_P301L_)4510 (or rTg4510) mouse has often been used. These mice contain a repressible form of human tau containing the P301L mutation restricted to the forebrain. It is likely that the hemizygous alterations of these genes contribute to the progressive age-related neurofibrillary tangles, neuronal loss, behavioural impairments and reduced neocortical network activity [24–26].

In the rTg4510 model, expressing the human tau transgene has significant effects on neurodegeneration and cognitive impairment [27, 28]. The unique aspect of this model is the ability to inactivate the gene and reduce tau expression by administering the tetracycline analogue doxycycline (DOX) [27, 29]. DOX-treated rTg4510 mice show reduced neurodegeneration and perform significantly better than their un-repressed counterparts in the Morris water maze task [25, 30, 31].

Much of the degeneration occurs in the hippocampus and cortex of rTg4510 mice, which are two of the most affected areas in patients [32, 33]. However, it has recently been shown that overexpression of the tau transgene in the hippocampus and cortex may not be sufficient to elicit significant neurodegeneration [34]. Nevertheless, there is still much that can be learned from this model regarding neurodegeneration using longitudinal studies with well-defined endpoints and appropriate controls.

Our aim in this study was to simultaneously record several different parameters in parallel cohorts of wild-type, tetracycline transactivator alone (tTA), Tg and Tg+DOX mice over the full time-course of pathological development to determine changes in sleep and EEG spectral power in rTg4510 mice occurring over time, in correlation with behavioural measures and terminal histology, and whether any observed changes could be inhibited by repressing the tau transgene.

## Materials and methods

### Transgenic mice

All procedures were approved by the Lilly UK Institutional Animal Care and Use Committee and were conducted in accordance with the Guide for Care and Use of Laboratory Animals (Institute of Laboratory Animal Resources, 1996) and the Animals (Scientific Procedures) Act 1986. All studies are reported in accordance with the ARRIVE guidelines for reporting experiments involving animals [35].

rTg4510 mice (129S6;FVB-Tg (Camk2a-tTA)1Mmay Tg (tet-o-MAPT*P301L)) were bred to be heterozygous for tetracycline transactivator under the Ca^2+^–calmodulin kinase II promoter and heterozygous for P301L tau to produce regulatable double transgenics as first described [25, 28] by Taconic (Germantown, USA). Three of the four produced lines were used in the present study: heterozygous tetracycline transactivator carriers but wild-type for tau (tTA), wild-type for both tTA and tau (WT) and both tTA carriers and tau carriers (Tg). A total of 100 male mice (25 WT, 25 tTA, 50 Tg) were used in the study. Mice were born within 3 weeks of each other from 39 breeding pairs.

All mice underwent baseline T-maze and open-field locomotor activity (ofLMA) evaluation between 9 and 12 weeks of age. Tg mice were randomised to ensure that an even distribution of high and low performers from the T-maze received doxycycline treatment. Of the 50 Tg mice, 25 were given DOX treatment beginning at 13 weeks of age (two 10 mg/kg bolus oral doses of doxycycline followed by Harlan Teklab base diet 2016 containing 200 ppm doxycycline for the duration of the experiment). These transgene-altered Tg mice were designated Tg+DOX.

**Fig. 1:**
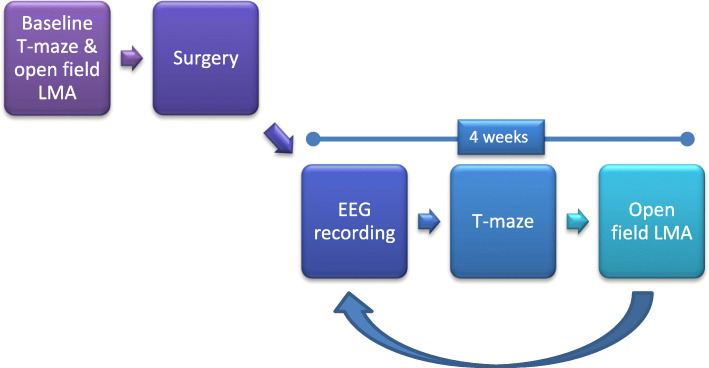
Experiment timeline. All mice underwent the same experiment timeline. Baseline T-maze and ofLMA testing occurred before surgery. After a recovery period, mice underwent 4-week cycles containing 2 weeks of EEG recording, T-maze and ofLMA evaluation

Studies were run in a 4-week cycle of tests—2 weeks of EEG recordings with ad libitum food followed by 2 weeks of food restriction and behavioural testing (T-maze and ofLMA) (Fig. 1). The first week of EEG recordings was used for acclimatisation and was therefore not analysed. Animals completed seven test cycles, unless they were removed for welfare reasons, until they reached approximately 46 weeks old, when they were euthanised.

### Surgical preparation

Mice underwent in-house surgery at 19.8–31.0 g and 11–17 weeks old. Subjects were anaesthetised and sedated (2% isoflurane in 100% oxygen, 0.1 mg/kg medetomidine HCl subcutaneous (SC) injection) and fitted with a cranial implant affixed to the skull by dental acrylic and cyanoacrylate. The implant consisted of five stainless steel screws for local field potential (LFP) electroencephalogram (EEG) recording (one frontal screw placed 2 mm anterior to bregma and 2 mm left of the sagittal suture line, one occipital placed 3 mm caudal from bregma and 3 mm right of the sagittal suture line, two stabilising screws and one ground screw over the cerebellum). Two Teflon-coated stainless steel wires were positioned under the nuchal trapezoid muscles for electromyogram (EMG) recording. Atipamezole (0.5 mg/kg, SC) was administered to reverse the medetomidine. Carprofen (5 mg/kg, SC) was administered pre-operatively, post-surgery and on the morning of the first post-operative day. Cefovecin (8 mg/kg, SC) was administered post-surgery.

### Housing environment

Following a 3-week recovery period, animals were housed individually in custom-designed cages. Cranial implants were connected to ultra-low-torque slip-ring commutators (Hypnion, Inc., Lexington, MA, USA) by a flexible tether to allow unrestrained movement. A 12-h light/dark cycle was maintained using white LEDs (35–40 lx) during the light period and red (> 680 nm) LEDs during the dark period, which enables infrared recording. Food (2916 diet or TD.120782 diet containing 200 ppm doxycycline, Envigo, UK) and water were available ad libitum, ambient temperature was 23 ± 1 °C and relative humidity averaged 50%. A digital video camera allowed remote visual monitoring.

### Behavioural testing

#### Open-field locomotor activity

A 40 × 40 × 30 cm Perspex arena was used to assess spontaneous locomotor activity during the light period but under complete darkness over 60-min trials. The mice were untethered, and movement within the arena was monitored using overhead infrared cameras (Sanyo VCV-3412P, Tracksys Ltd., UK). Cameras fed into a Quad compressor unit (Sanyo VDM-801P, Tracksys Ltd., UK) which relayed data to a computer running the image analysis software (Ethovision XT v8.5, Noldus, Netherlands) to calculate distance moved.

#### T-maze rewarded alternation

Discrete-trial rewarded alternation was tested using a semi-automated T-maze (Apogee Engineering Analysis Solutions, Norwich, UK) as described previously [27]. This apparatus was constructed of matte black, 8-cm-wide Perspex with 20-cm-high transparent Perspex walls. The external lengths of maze edges were 86 cm (choice end), 105 cm (return arm) and 22.5 cm (delay end). The centre arm was 83 cm in length, and a door was located 63 cm from the choice point forming a holding area at the base of the start arm. The entry of an animal into specific areas of the maze was detected using infrared beam breaks and passed to a microcontroller (Arduino Mega 2560, RS Components, UK). Matlab programs automatically controlled the maze doors and test procedure, allowing it to run without human intervention. Rewards were delivered by three pellet dispensers, one located at the end of each reward arm and a third in the delay/holding area of the maze to encourage return of the animal to the starting point for the subsequent test phase or trial. Mice were trained following a two-stage protocol: forced alternation training and discrete-trial rewarded alternation testing. During the forced alternation training stage, mice were released from the holding area at the base of the T-maze, allowed to run along the centre arm and forced to turn toward one of the reward areas to receive a sucrose pellet reward. Mice then returned to the holding area to collect a second reward pellet, after which another forced trial was initiated. At this stage, a 0-s inter-trial interval was used, and mice were trained for a maximum of 60 trials or 30 min daily. Once most mice were performing 40 training trials or more in a session, mice were moved on to the discrete-trial rewarded alternation protocol. Each trial consisted of two phases—a sample phase and a test phase. During the sample phase, mice were forced to turn toward the left or right arm and return to the starting/holding area. Two reward pellets were collected along the way, one at the end of the choice arm and one in the holding area. During the test phase of each trial, mice could choose between the two arms of the T-maze but were rewarded when visiting the novel arm only (i.e. arm not explored during the sample period). Forced left or right allocations during the sample phase were pseudo-randomised with no more than three consecutive sample runs to the same side. Mice could run for a maximum of 20 trials or 30 min daily over 3 days of testing. A 2-s inter-trial interval and a 5-s sample-to-test delay were used. The percentage of correct choices (primary outcome measure: number of correct choice/number of trials) as well as the choice latency (secondary outcome measure) were recorded and calculated for each animal.

### Histology

Animals were terminally anaesthetised with pentobarbital and cardiac perfused with saline. Brains were then removed and weighed. Brain samples were cut into two coronal blocks using an adult mouse coronal brain matrix (ASI Instruments Inc., Warren, MI, USA) and processed using the Tissue TEK VIP processor (GMI Inc., Ramsey, MN, USA) before being embedded in paraffin wax for coronal brain sectioning. Serial sections (6 μm) were taken using HM 200 and HM 355 rotary microtomes (Thermo Scientific Microm, Germany). Immunohistochemistry was performed using a primary antibody for tau phosphorylated at serine 409 (PG-5, 0.11 μg/ml from Peter Davies; Albert Einstein College of Medicine, Bronx, NY, USA) and NeuN (1:500 from Millipore; MAB377) as previously described [29]. Stained sections were digitised using the Scanscope XT slide scanner (Aperio, CA, USA) at × 20 magnification. Imagescope software (version 11.1.2.760; Aperio) was used to view the digitised tissue sections and delineate the regions of interest (ROIs) which included the hippocampus, cortex (visual and somatosensory areas), lateral hypothalamic area (LH) and ventromedial hypothalamic area (VMH: ventromedial nucleus, arcuate nucleus and median eminence). PG-5-positive tau pathology was quantified in these ROIs using a pathology scoring algorithm [1–5] by a pathologist blinded to treatment. Due to heavy staining of some sections, a different approach was used to quantify atrophy. The thickness of the hippocampus and cortex were digitally measured to create an indication of atrophy, averaged across the left and right sides of the brain.

### Data collection, sleep staging and statistical analysis

The SCORE2004™ bioassay facilitated the acquisition of multiple concomitant physiological measurements from 30 animals simultaneously. Validation of the SCORE2004™ technology has been previously described [36–38]. EEG signals, recorded as the differential between front and back contralateral skull screws, were amplified 10,000×, bandpass filtered at 1–300 Hz and digitised at 400 Hz (Grass Corp., Quincy, MA, USA). EMG signals were amplified 20,000×, bandpass filtered at 10–100 Hz, integrated based on the root mean square (RMS) and recorded as arbitrary units per 10-s epoch. Arousal states were then classified on-line as NREM sleep, REM sleep, wake or theta-dominated wake in 10-s epochs using EEG period and amplitude feature extraction and ranked membership algorithms. Sleep bouts were determined as a minimum of 3 consecutive epochs of NREM, or 2 consecutive epochs of REM. Individually optimised EEG-arousal-state templates and EMG criteria differentiated states of arousal. Sleep data are presented as least squares means. Spectral frequency bands were delta (0.1 to 4 Hz), theta (5.1 to 9 Hz), alpha (9.1 to 12 Hz) and beta (12 to 20 Hz) based on internal and external data [39–44]. Total power was selected as 0.1 to 30 Hz. Many of the analyses were separated into light or dark period to reflect significant periods of sleep and wake respectively. An average of one full week of data for each 12-h light or dark period was used from each animal every 4 weeks.

Data quality control was assured by regular inspection of EEG blinded to the treatment group by expert data analysts using a proprietary suite of programs (SCOREVIEW™, Hypnion, Inc., Lexington, MA, USA). Signal quality checks were performed weekly and non-physiological artefacts such as signal-clipping or flat signal were contemporaneously removed from the analysis. Data were excluded from further analysis based on signal quality. If artefacts exceeded 5% of epochs, that week of data was excluded. Cable-based issues could be fixed by cable replacement on a weekly basis during cage change to minimise disruption. A minority of animals suffered ongoing artefacts due to damage to the electrodes during surgery and were excluded from all analyses. Sample size, after quality control, for each treatment group at each time point ranged between 12 and 23. EEG amplitude reduced over time in some rTG4510 mice, particularly after week 36, to the extent that scoring became unreliable. The EEG and EMG data for these affected animals were omitted from the analysis, thus reducing sample size in this treatment group at weeks 40 and 44 to *n* = 4–7.

For video locomotor activity (video LMA), the feed was converted to greyscale with 720 × 480 pixels per frame. The difference between each frame was calculated as the sum of the total number of pixels that changed relative to the previous frame. A minimum threshold of at least 10-pixel values changed per frame was necessary to limit the effects of video compression.

Statistical analysis of the longitudinal measures was conducted using a repeated measures analysis of variance and reported as least square (LS) means. For all responses, the model used strain, animal age, and the interaction of strain by age to estimate the mean response difference between each of the 6 pair-wise strains at each of the 7 animal age time points. In addition, ofLMA included the location of the experiment in the model due to known effects. A post hoc Tukey-Kramer multiple comparison adjustment was used to control for the type I error rate based on the 42 pair-wise comparisons of interest. An unstructured covariance matrix was used to model the correlation of observations on the same animal. The bouts and spectral power outcomes were analysed on the LOG scale, as well as the T-maze measure average choice latency and the ofLMA total distance response. Estimates have been back transformed, and differences will be interpreted as multiplicative.

Tau pathology and hippocampal thickness were analysed by ANOVA and Tukey post hoc test. A correlation analysis compared average bilateral cortex and hippocampus thickness with the last observation per animal during the dark period of the electrophysiological and behavioural measures. Actual values and Spearman residuals were analysed across all 4 treatments.

## Results

### EEG spectral power declines over time in rTg4510 mice

**Fig. 2:**
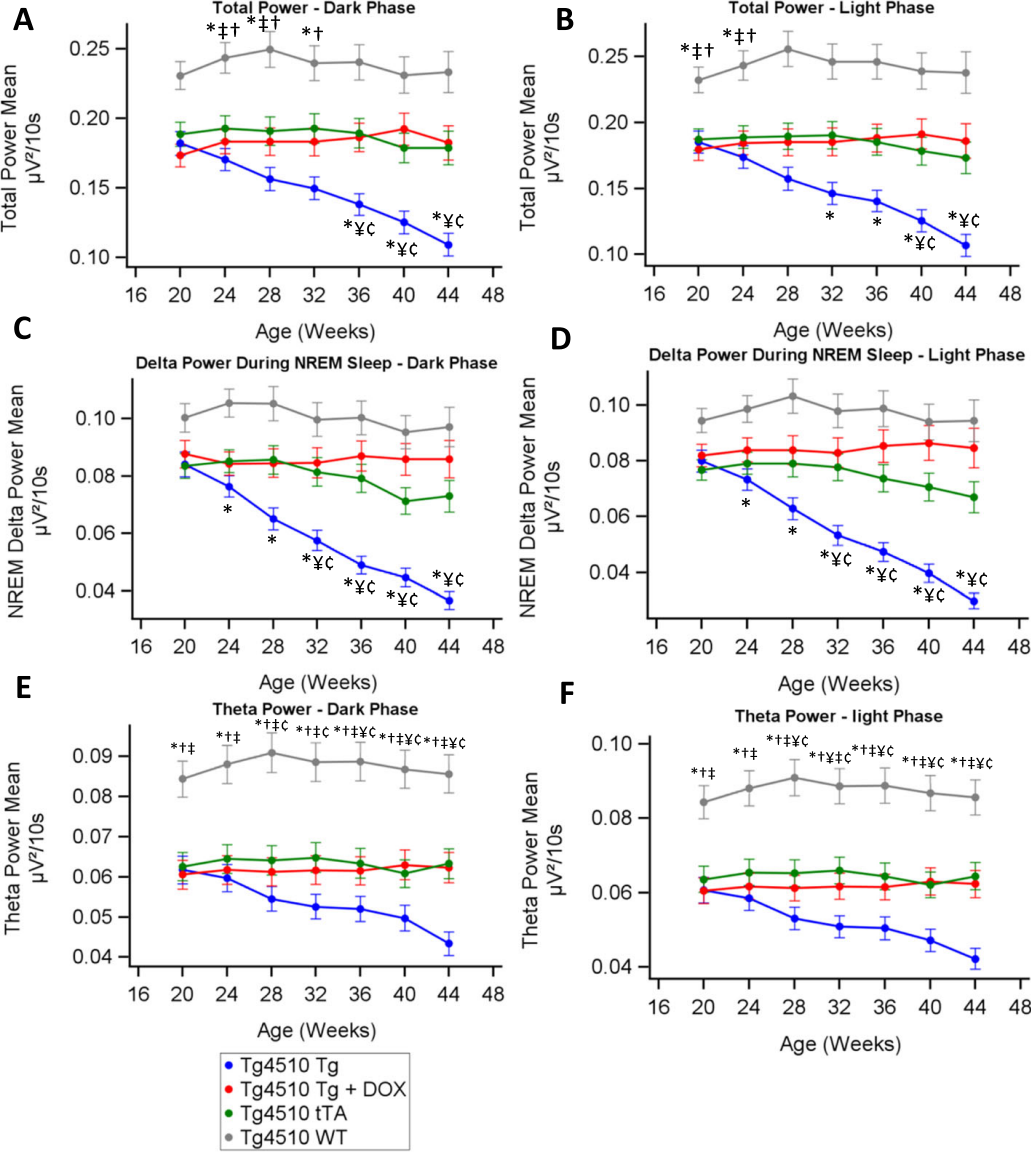
EEG spectral power decreases over time in rTg4510 mice. Average total EEG power from 1-week recording periods every 4 weeks in the dark (**a**) and light (**b**). Average delta power during NREM sleep from 1-week recording periods every 4 weeks in the dark (**c**) and light (**d**). Average theta power from 1-week recording periods every 4 weeks in the dark (**e**) and light (**f**). Asterisks (*) denote statistical significance (*p* < 0.05) at the time point between WT vs Tg, ^†^WT vs Tg+DOX, ^‡^WT vs tTA, ^¥^Tg vs Tg+DOX, ^¢^Tg vs tTA and ^¤^Tg+DOX vs tTA. Any statistical significance in groups compared with time is described in the text and not shown in the graphs (*n* = 12–23; *p* ≤ 0.05)

We continuously measured EEG parameters for 1 week in every 4 weeks. Total power (0.1 to 30 Hz) independent of sleep state during the light and dark cycles stayed constant over the testing period in WT, tTA and Tg+DOX groups (*p* > 0.01, Fig. 2a, b). Total power in the Tg group declined on a weekly basis from week 20 becoming significantly lower than all other groups from week 36 in the dark period and week 40 in the light period. Total power was higher in the WT group compared to all other groups at weeks 24 to 28 in both the dark and light periods. EEG spectral power changed over time in different treatment groups (supplementary figures 1A-D) so we isolated the delta and theta bands for further analysis. NREM delta power (0.1 to 4 Hz) declined over time in the Tg group (*p* < 0.01, Fig. 2c, d). There were no initial significant differences between WT and tTA or Tg in NREM delta power. Delta power in the Tg+DOX group was not different from the tTA group at any point. Theta power (5.1–9 Hz) declined over time in the Tg group (*p* < 0.01, Fig. 2e, f). Theta power significantly differed between the WT and all other groups at all time points. Theta power in Tg+DOX was not different from the tTA group at any point. Doxycycline treatment fully prevented the decline in total power, including the NREM delta and theta bands.

### rTg4510 mice have altered sleep architecture

**Fig. 3:**
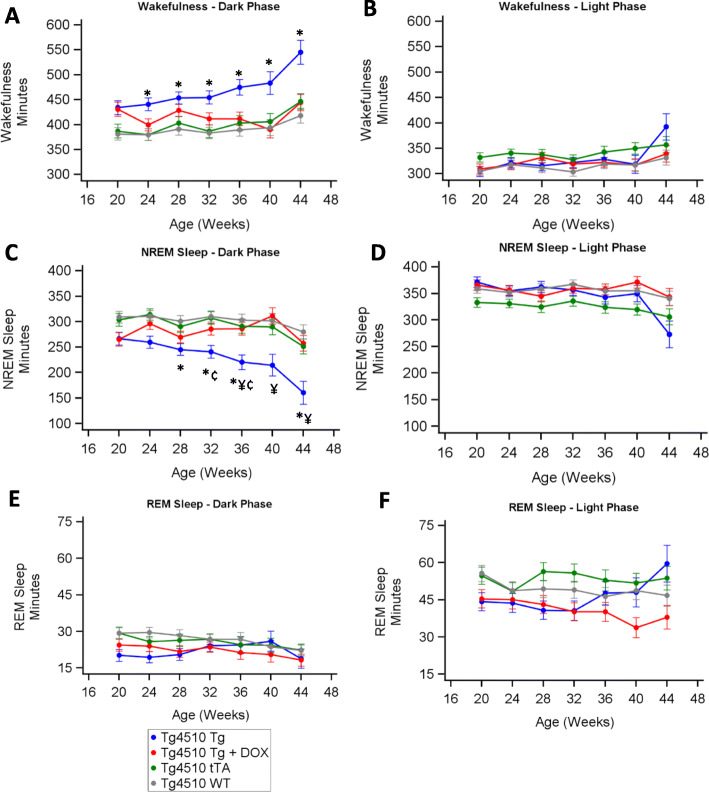
Sleep architecture changes over time in rTg4510 mice. Least squares means from 1-week recording periods every 4 weeks in the dark and light periods for wakefulness (**a**, **b**), NREM sleep (**c**, **d**) and REM sleep (**e**, **f**), respectively. Asterisks (*) denote statistical significance (*p* < 0.05) at the time point between WT vs Tg, ^†^WT vs Tg+DOX, ^‡^WT vs tTA, ^¥^Tg vs Tg+DOX, ^¢^Tg vs tTA and ^¤^Tg+DOX vs tTA. Any statistical significance in groups compared with time is described in the text and not shown in the graphs (*n* = 12–23; *p* ≤ 0.05)

Time spent in wakefulness during the dark period increased in the Tg group relative to WT from week 24 (Fig. 3a). During the dark period, both tTA and Tg+DOX showed similar amounts of wakefulness compared with WT at all time points. Levels of wakefulness were similar for all groups during the light period (Fig. 3b). Time spent in NREM sleep during the dark period declined in the Tg group relative to all other groups from week 28 (Fig. 3c). The decline in NREM sleep in the Tg group was fully prevented by doxycycline treatment as demonstrated by a correlation between NREM sleep and brain atrophy (*r*_s_ = 0.39, *p* < 0.001; Table 1 & supplementary figure 2A). During the light period, time spent in NREM sleep was similar across all groups (Fig. 3d). Total time spent in REM sleep was unchanged for all strains during the light and dark periods (Fig. 3e, f).

**Table 1:**
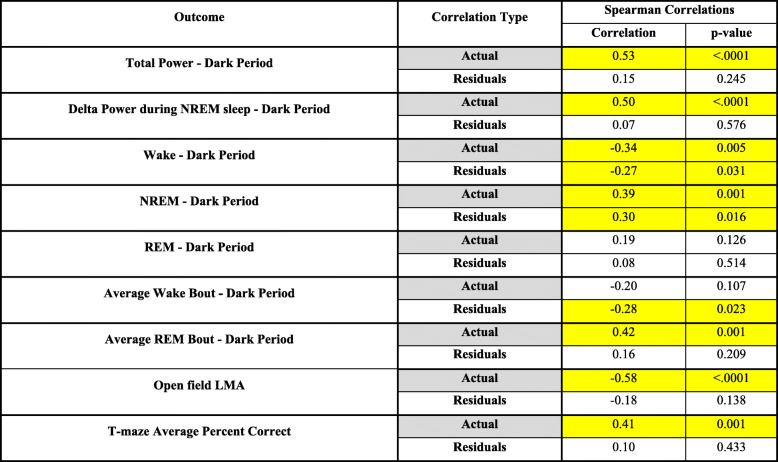
Correlations between physiology and atrophy. Statistical correlations between sum bilateral hippocampus and cortex thickness vs final time point average for the main electrophysiological and behavioural outcomes. Rows highlighted in yellow denote statistical significance (*n* = 12–18; *p* ≤ 0.05)

### The influence of transgene strain on natural circadian rhythms

**Fig. 4:**
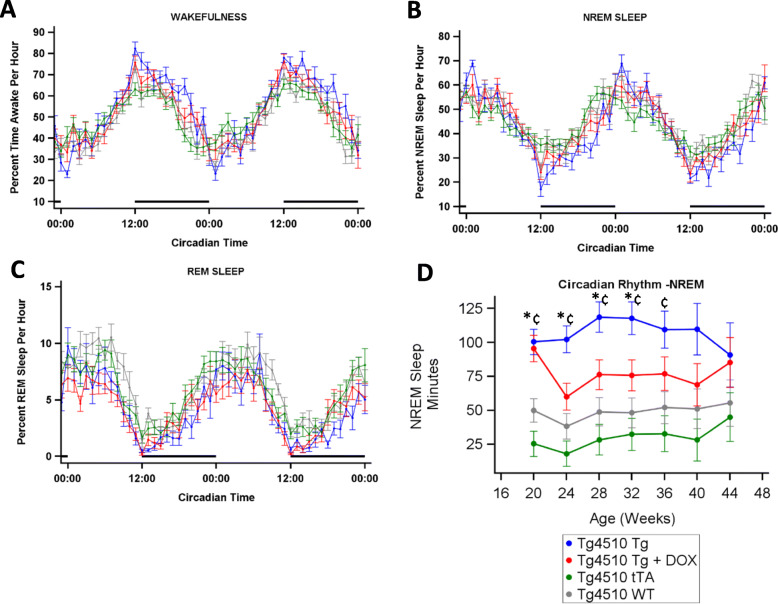
Circadian wake and sleep differences between strains of mice. Percent time in wakefulness, NREM sleep and REM sleep from a 48-h recording period at 32 weeks of age (*n* = 9–13) (**a**, **b**, and **c** respectively). Average difference in NREM sleep between light and dark period from 1-week recording periods every 4 weeks (*n* = 12–23) (**d**). Asterisks (*) denote statistical significance (*p* < 0.05) at the time point between WT vs Tg, ^†^WT vs Tg+DOX, ^‡^WT vs tTA, ^¥^Tg vs Tg+DOX, ^¢^Tg vs tTA and ^¤^Tg+DOX vs tTA

We observed circadian variations in wake, NREM and REM sleep between the groups (Fig. 4a–c). Consequently, we quantified the light/dark variability over time for NREM sleep (Fig. 4d). The Tg group had a larger light/dark cycle amplitude in the sleep parameters compared with the WT and tTA groups from week 20 until week 32 or 36 respectively (*p* < 0.05, Fig. 4d). DOX treatment from 13 weeks of age did not significantly alter the function. Within the same strain, the ratios of the sleep components remained constant over time (*n* = 12–23; *p* ≤ 0.05).

### Changes in sleep continuity over time

**Fig. 5:**
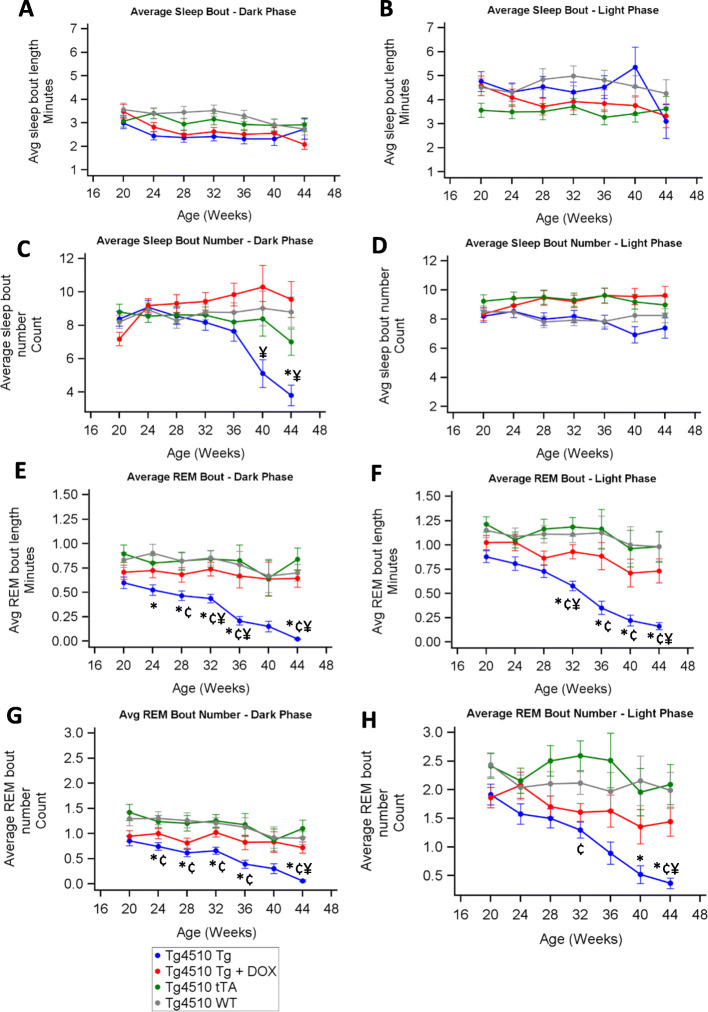
Sleep continuity changes over time in rTg4510 mice. Average sleep (NREM plus REM) bout length (> 30 s duration) during sleep from 1-week recording periods every 4 weeks in the dark (**a**) and light (**b**). Average sleep bout number from 1-week recording periods every 4 weeks in the dark (**c**) and light (**d**). Average length of REM sleep bout length (> 20 s duration) from 1-week recording periods every 4 weeks in the dark (**e**) and light (**f**). Average length of REM sleep bout number from 1-week recording periods every 4 weeks in the light (**g**) and dark (**h**). Asterisks (*) denote statistical significance (*p* < 0.05) at the time point between WT vs Tg, ^†^WT vs Tg+DOX, ^‡^WT vs tTA, ^¥^Tg vs Tg+DOX, ^¢^Tg vs tTA and ^¤^Tg+DOX vs tTA

Average sleep bout length remained similar for all groups in both light and dark across all time points (Fig. 5a, b). The number of sleep bouts during the dark period declined in the Tg group compared with Tg+DOX by week 40 (Fig. 5c). The sleep bout number was not different between tTA, Tg+DOX and WT groups during the dark period, nor between any of the groups in the light period (Fig. 5d). REM bout length during both the dark and light periods decreased over time in the Tg compared to the other three groups (Fig. 5e, f). The number of REM bouts during both the dark and light periods also declined over time in the Tg group compared to the other groups (Fig. 5g, h). From this analysis, it appeared that the REM sleep of the Tg group appeared fragmented beyond the criteria for REM bout detection. This led to a divergence between total REM time and REM continuity. We looked at the distribution of all REM sleep episodes in the Tg group and the majority were only 1 epoch long (10 s).

### Behaviour changes in rTg4150 mice

**Fig. 6:**
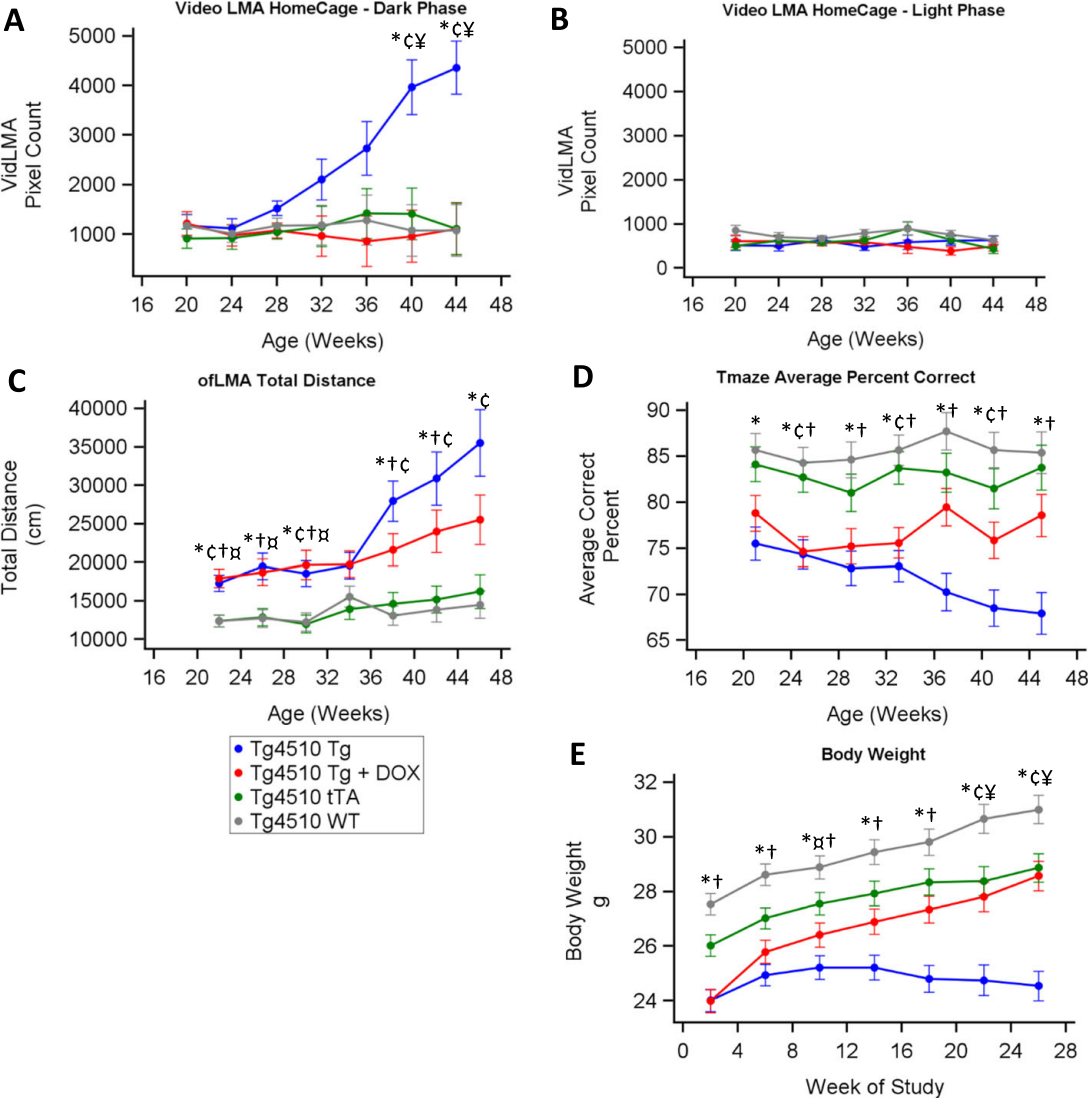
Changes in behaviour over time in rTg4510 mice. Relative distance moved during the dark (**a**) and light (**b**) periods in the home cage environment while undergoing EEG measurements. Distance moved during 1-h recording periods every 4 weeks in an open-field locomotor test (**c**). Average percent correct in the rewarded alternation T-maze (**d**). Body weight (**e**). Asterisks (*) denote statistical significance (*p* < 0.05) at the time point between WT vs Tg, ^†^WT vs Tg+DOX, ^‡^WT vs tTA, ^¥^Tg vs Tg+DOX, ^¢^Tg vs tTA and ^¤^Tg+DOX vs tTA

During the dark period, video LMA significantly increased in the Tg group from week 32 vs the other groups (Fig. 6a). Tg+DOX and tTA groups were similar to WT across all time points indicating that doxycycline completely prevented this change in video LMA. Activity during the light period remained low across all the time points demonstrating the light/dark dependence of the video LMA increase (Fig. 6b). During ofLMA, Tg was significantly higher than WT and tTA groups at most time points (Fig. 6c).

In the T-maze studies, the Tg group showed significant reductions in the percentage of correct decisions compared to WT which declined further over time (Fig. 6d). Doxycycline treatment prevented the decline, but this did not reach statistical significance in the model (*p* = 0.06 at week 45). The tTA group performed significantly better than the Tg group at weeks 25 and 33–45.

The body weight of the Tg group was significantly lower than that of the WT group at all time points and lower than that of the tTA group from week 10 (Fig. 6e). Tg+DOX was significantly lower in weight compared with WT between weeks 2 and 10, but then increased to become significantly higher than Tg by week 22.

### Doxycycline inhibited tau and atrophy of cortical, hippocampal and hypothalamic areas in rTg4510 mice

**Fig. 7:**
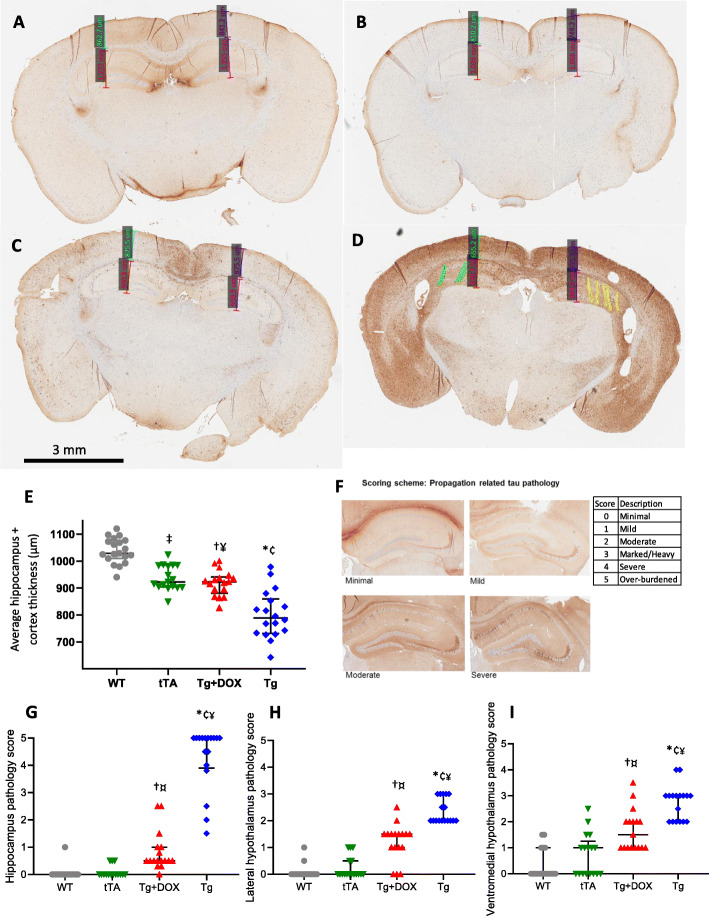
Propagation of tau and atrophy. Representative brain sections with measures of hippocampus and cortex thickness as an indicator of atrophy in WT (**a**), tTA (**b**), Tg+DOX (**c**) and Tg (**d**) mice. Calculated average bilateral hippocampus and cortex thickness (**e**). An additional scoring scheme of tau propagation with PG-5-positive cell expression (brown) and NeuN (blue) in the hippocampus (**f**) with corresponding pathology score. Pathology scores for the hippocampus (**g**), lateral hypothalamus (**h**) and ventromedial hypothalamus (**i**). Asterisks (*) denote statistical significance (*p* < 0.05) between WT vs Tg, ^†^WT vs Tg+DOX, ^‡^WT vs tTA, ^¥^Tg vs Tg+DOX, ^¢^Tg vs tTA and ^¤^Tg+DOX vs tTA

Pathological examination at week 46 shows increased PG-5 staining and atrophy in Tg mice compared with WT, tTA or Tg+DOX (Fig. 7a–d). The average bilateral cortex and hippocampus thickness was used as a measure of atrophy. The Tg, Tg+DOX and tTA groups had significantly reduced thickness compared with WT (Fig. 7d, ANOVA, *F* (3, 67) = 49.4, *p* < 0.001). Doxycycline significantly attenuated atrophy compared to the Tg group. Additional analysis showed the Tg and Tg+DOX groups had significantly higher tau pathology in the hippocampi compared to the tTA or WT groups (Fig. 7f, g, ANOVA, *F* (3, 65) = 136.7, *p* < 0.001). The Tg+DOX group had significantly less tau than the Tg group. Similar to the hippocampus, we observed significantly higher tau in both the lateral hypothalamic and the ventromedial hypothalamic areas in the Tg and Tg+DOX groups compared to the tTA or WT groups (Fig. 7h, i, ANOVA, *F* (3, 65) = 84.3, *p* < 0.001 and *F* (3, 65) = 38.9, *p* < 0.001 respectively). In both hypothalamic areas, the Tg+DOX group had significantly less tau than the Tg group.

### Correlations between physiology and atrophy

Atrophy was highly correlated with many of the final time point outcomes, with statistical significance between actual values indicating a difference in the group means (Table 1). NREM sleep during the dark period was significantly correlated to atrophy (supplementary figure 2A). Correlations between the Spearman’s residuals identify that there were far fewer significant correlations within the underlying groups in any of the measures when compared to atrophy. For example, the actual value analysis for ofLMA vs atrophy was significantly correlated, but when adjusted for treatment effect, the residual value analysis shows no significant difference (Table 1 and supplementary figure 2B).

## Discussion

We show that a model of tauopathy displays significant longitudinal neurological changes manifested by altered sleep architecture, decreased spectral power and decreases in spatial working memory. Bi-transgenic rTg4510 (Tg) male mice differed from non-transgenic (WT) controls with regard to spectral power, NREM sleep, wake bouts, REM sleep bouts, open-field LMA and T-maze accuracy. Repressing tauopathy progression with DOX from 13 weeks of age was able to prevent or attenuate most of the longitudinal EEG changes. DOX was sufficient to reverse hyperactivity and T-maze accuracy back to the levels found in the tTA or WT mice. DOX prevented severe tau pathology, and the average cortex and hippocampus thickness resembled that of the tTA group. These data demonstrate that the progressive tauopathy in the Tg mice is the cause of the majority of the changes in sleep and EEG.

### EEG changes

Spectral power decreased linearly from 24 weeks of age in the Tg group. There were significant reductions in spectral power at an earlier time point than wake or NREM in Tg vs control mice. In addition, wake and NREM sleep correlated significantly with cortical and hippocampal atrophy, even when values were adjusted for treatment groups. Though we saw correlations in the actual values between atrophy and many other measures, there were fewer correlations within the underlying groups.

Both total power and delta power within treatment group were similar for the light and dark periods. The WT group experienced higher total power than tTa and Tg groups suggesting that the presence of the tTA transgene conferred a difference in EEG power. By 32 weeks of age, the Tg group had significantly reduced total power and delta and theta power, a likely consequence of the progressive tauopathy. At the same time, there was an increase in wakefulness and a decrease in NREM sleep in the Tg group relative to the other groups. Further investigations of sleep continuity revealed that the Tg group had a reduced number of NREM and REM bouts over time during the dark period relative to other groups, with a corresponding increased number of wake bouts. This was a similar finding to that reported in the P301S mouse [15]. Strikingly, we show that REM bouts were significantly shorter in the Tg group suggesting that they were unable to maintain continuity of REM sleep. This likely reduced the potential benefits of this sleep stage during the later parts of the study, potentially impacting on cognitive behaviour [45]. Further analysis of sleep state transitions could reveal additional insight into the effects of these changes.

The changes we have observed in this study match some of those reported in other tau overexpression models and amyloid-related models. For example, the decline in REM sleep and increased wake occur in the P301S model but at a later time point, which may be related to the slower pathology reported in this strain [15]. Other studies have shown effects on sleep and spectral activity in different models at different time points [21–23, 46, 47], but this is the first study to carry out continued measurements throughout the pathology time-course and examine these simultaneously in a longitudinal fashion in the same animals. In addition, the use of DOX has enabled us to demonstrate that these effects are directly related to tau-mediated neurodegeneration.

We observed a higher NREM sleep-based peak-to-peak circadian rhythm amplitude in the Tg group relative to the other strains. The circadian rhythm amplitude did not change over time despite a reduction in NREM sleep during the dark period and no corresponding decrease in NREM sleep during the light period. Our data complements previous research showing that tauopathy mice exhibit longer, more fragmented free-running periods than controls in both the P301L [48] and P301S models at 11 months of age [15]. These data illustrate that tauopathy can directly influence certain aspects of circadian physiology while others do not change with increased expression.

It is possible that DOX can impact sleep parameters although we are not aware of any literature measuring EEG spectral power on tTA or WT mice administered DOX. We control for any acute effects of DOX by starting treatment prior to surgery and first measuring EEG at a time point where there were no differences between DOX-treated and untreated mice.

### Histopathological changes

At 46 weeks, rTG4510 mice had significant degeneration in the hippocampus and cortex. DOX prevented severe tau pathology, and the average cortex and hippocampus thickness resembled that of the tTA group. The tTA group displayed reduced spectral power and some hippocampal atrophy compared to WT mice. EEG power did not change over the course of the study indicating that the primary cause for this occurs prior to the start of the study and is potentially due to the expression of the tTA transgene. Atrophy in the dentate gyrus has previously been reported for tTA mice and is present as early as 2 months of age [49]. The lower spectral power may be a consequence of the hippocampal atrophy. Some phenotypes observed in rTg4510 mice have recently been claimed to be due to disruptive insertion of the tau and tTA transgenes into unintended areas of the mouse genome rather than due to overexpression of the tau transgene itself into cortex and hippocampal regions [34]. Multiple copies of the tau transgene are inserted into the Fgf14 promotor and gene, plus multiple copies of the tTA transgene disrupt or delete Vipr2, Wdr60, Esyt2, Ncapg2 and Ptprn2 genes. Here we account for the tTA transgene disruption by including a tTA group not expressing P301L tau and the use of doxycycline to inhibit P301L tau expression. We identified some differences between groups across most measured outcomes and showed that the tTA and WT groups differ little in sleep architecture, sleep continuity and behaviour. Inactivating mutant tau from 13 weeks of age by administering DOX produced significant protection against the changes in sleep architecture, sleep continuity and behaviour in the Tg group, providing evidence that the genome disruptions proposed by [34] do not create the full biological tauopathy phenotype. By using appropriate controls, the rTg4510 mouse can still be utilised to investigate potential tauopathy-related therapeutics.

Concomitant with the cortical and hippocampal degeneration, we observed an increase in lateral and ventromedial hypothalamic tau burden relative to all other groups. Our data is in agreement with previous research showing increased tau in the SCN and dorsomedial hypothalamus of rTG4510 mice [48, 50]. The LH contains neurons with key neurotransmitters involved in sleep and wake namely melanin-concentrating hormone, GABA and orexin [51]. Neurons in both the LH and VMH regulate homeostatic control of feeding and stress [51–53]. It is possible that increased tau burden in these hypothalamic areas is in part responsible for the corresponding reduction in sleep or body weight and increase in open-field LMA between treatment groups. Altering the duration of DOX treatment would be required to further explore this finding.

### Behaviour changes

Our data are in agreement with previous reports showing reduced body weight, increased locomotor activity during the dark phase and increased tau in the hypothalamus of rTG4510 mice compared to WT [50]. Further, it is interesting to note that the tTA group had higher cortical and hippocampal atrophy compared to the WT group but no difference in tau burden across any brain area analysed. This further implicates the role of tTA in non-pathological neuronal network dysfunction.

The changes in open-field locomotor activity and T-maze performance we observe are similar to those previously reported [27]. In our experiments, the differences between the Tg and Tg+DOX groups in the open-field LMA or T-maze did not reach statistical significance but there were robust, DOX-sensitive, changes in home cage LMA during the dark period. In the open-field environment, a clear DOX insensitive component was identified from the start of the study. This was not present in the tTA group and, given the DOX insensitivity, is unlikely to be tau related in concordance with previous reports [54]. We hypothesise that measuring LMA during a more stressful environment during the animal’s sleep period, such as the open-field LMA test, may involve additional components to those in a less stressful home cage environment. The data we present shows that DOX treatment normalises cognition in rTg4510 mice toward the wild-type. While no rodent model can adequately express all the symptoms of Alzheimer’s disease, we present useful translatable information regarding changes in sleep architecture, EEG, behaviour and cognition that are dependent on, and correlated to, tau pathology. Back-translated assays of AD and FTD are vital for successful drug discovery [55], but care must be taken in overinterpreting findings in mice models. For example, we did not observe marked sleep fragmentation commonly reported in AD and FTD [56, 57]. Data from this study provides further evidence that the rTg4510 tauopathy model can be useful in the investigation of tau-related functional deficits.

We suggest that any studies using the rTg4510 strain to investigate tauopathy should start by 13 weeks of age or earlier. As the Tg group aged beyond 40 weeks, most measured outcomes were severely affected. Histological analysis shows severe neurodegeneration, which is likely to be irreversible by any pharmacological treatment at this point. It has recently been reported that partial reduction of microglia by PLX3397 does not affect tau pathology in aged rTg4510 mice [58]. However, due to the severe pathology at the time point they began the treatment, it may have been too late to affect the existing tau-dependent neurodegeneration. In the current study, at ages above 40 weeks, it became difficult to discriminate between sleep stages due to an overall reduction in the amplitude of the EEG signal. AD patients tend to drift in between states of sleep and wake more easily [6, 59], and the identification of NREM and wake is less easily defined [6, 60]. Similar to AD patients, we observe a strong reduction in slow wave activity that precedes other deficits and is associated with tau deposition [14, 61].

Measurement of EEG power is a cheap and easily accessible method to study neurodegenerative disease. Both dementia and normal ageing display reduced EEG power [62], likely reflecting the breakdown of neuronal networks. Which areas of the brain, if any, first display network discontinuity during ageing or disease requires further research. Despite clear limitations around translatability, preclinical mouse models can still be useful to gain mechanistic understanding of tau-driven effects on brain function.

## Conclusions

In this study, we showed EEG spectral power, sleep architecture and sleep continuity are reduced over time in rTg4510 mice, relative to controls. Measures of locomotor activity were increased over time. Spectral power, in particular slow wave activity, reduced before changes in sleep architecture in rTG4510 mice relative to controls. All groups maintained a stable peak-to-peak circadian rhythm amplitude over time, though the rTg4510 group had a larger light/dark variability than wild-type controls. Wake and NREM sleep correlated significantly with hippocampal atrophy. We used 3 control groups to ensure our conclusions were within the appropriate context of the tauopathy model. We identify interesting parallels between altered sleep and circadian rhythms in rTg4510 mice compared with tau-related effects on sleep architecture in AD and FTD patients.

## Supplementary information

**Additional file 1: Supplementary figure 1.** spectral power. EEG spectral power from a 1-week recording period at 22 weeks of age or earlier in the dark (A) and light phase (B). EEG power from a 1-week recording period at 40 weeks of age or later in the dark (C) and light phase (D). Spectral frequency bands were delta (δ, 0.1 to 4 Hz), theta (θ, 5.1 to 9 Hz), alpha (α, 9.1 to 12 Hz) and beta (β, 12 to 20 Hz).

**Additional file 2: Supplementary figure 2.** correlation of NREM sleep or ofLMA vs atrophy. Correlation of atrophy vs NREM sleep; actual values (A) and residuals (B). Correlation of atrophy vs log distance in open field LMA; actual values (C) and residuals (D). Small symbols represent individual subjects. In the correlations with the actual values, the large symbols represent the average of the treatment group. Spearman’s correlation calculations in Table 1.

## Data Availability

The datasets used and/or analysed during the current study are available from the corresponding authors on request. An abstract titled “tau dependent temporal changes in EEG in male Tg4510 mice” presented at the 13th International Conference AD/PDTM (Vienna, Austria, March 29 to April 2, 2017), containing part of the data can be found at the following: [63].

## Abbreviations

AD: Alzheimer’s disease
ANOVA: Analysis of variance
DOX: Doxycycline
EEG: Electroencephalogram
EMG: Electromyogram
FTD: Frontotemporal dementia
HCl: Hydrochloric acid
LFP: Local field potential
LH: Lateral hypothalamic area
LMA: Locomotor activity
NeuN: Antibody for NEUronal Nuclei clone A60
NREM: Non-rapid-eye-movement sleep
ofLMA: Open-field locomotor activity assay
REM: Rapid-eye-movement sleep
RMS: Root mean square
ROI: Region of interest
rTg4510: Repressible rTg(tet-o-Tau_P301L_)4510
SC: Subcutaneous
Tg: rTG4510 with both tTA and tet-o-Tau_P301L_
Tg+DOX: rTG4510 with both tTA and tet-o-Tau_P301L_ but expression reduced with doxycycline at 13 weeks of age
tTa: Tetracycline transactivator carriers but wild-type for tau
VMH: Ventromedial hypothalamic area
WT: Wild-type for both tTA and tau
