# Supplementary material for: Longitudinal changes in EEG power, sleep cycles and behaviour in a tau model of neurodegeneration
Source: Alzheimers Res Ther. 2020 Jul 15;12:84. doi: 10.1186/s13195-020-00651-0 (PMC7364634; doi:10.1186/s13195-020-00651-0)
Supplement: Supplementary file 1 — Additional file 1: Supplementary figure 1. spectral power. EEG spectral power from a 1-week recording period at 22 weeks of age or earlier in the dark (A) and light phase (B). EEG power from a 1-week recording period at 40 weeks of age or later in the dark (C) and light phase (D). Spectral frequency bands were delta (δ, 0.1 to 4 Hz), theta (θ, 5.1 to 9 Hz), alpha (α, 9.1 to 12 Hz) and beta (β, 12 to 20 Hz). [file 13195_2020_651_MOESM1_ESM.pdf]

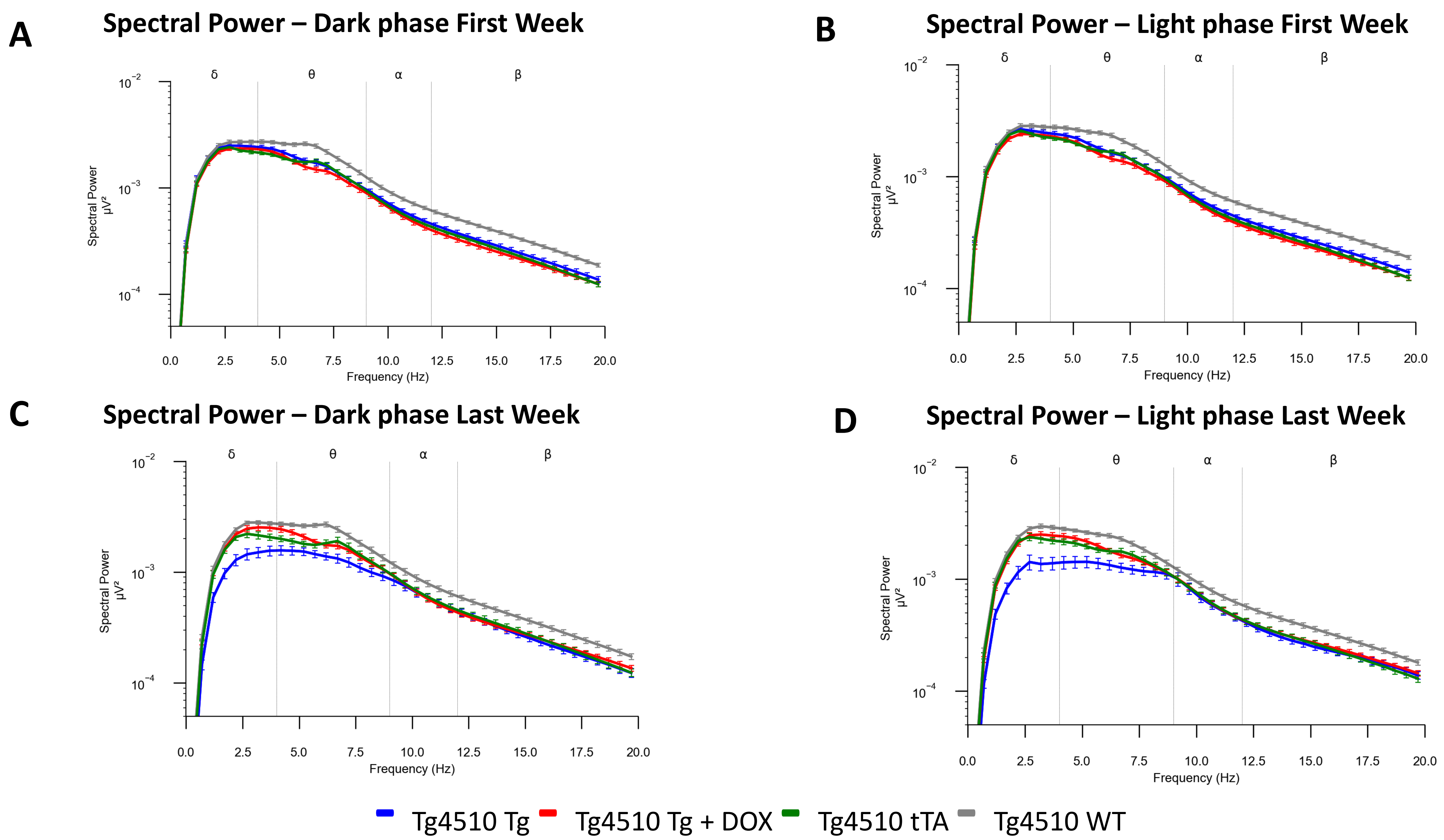
A
Spectral Power – Dark phase First Week
B
Spectral Power – Light phase First Week
C
Spectral Power – Dark phase Last Week
D
Spectral Power – Light phase Last Week
δ
θ
α
β
Spectral Power
μV²
10^{-2}
10^{-3}
10^{-4}
0.0
2.5
5.0
7.5
10.0
12.5
15.0
17.5
20.0
Frequency (Hz)
Tg4510 Tg
Tg4510 Tg + DOX
Tg4510 tTA
Tg4510 WT
