# Supplementary figures and images for: Longitudinal changes in EEG power, sleep cycles and behaviour in a tau model of neurodegeneration
Source: Alzheimers Res Ther. 2020 Jul 15;12:84. doi: 10.1186/s13195-020-00651-0 (PMC7364634; doi:10.1186/s13195-020-00651-0)

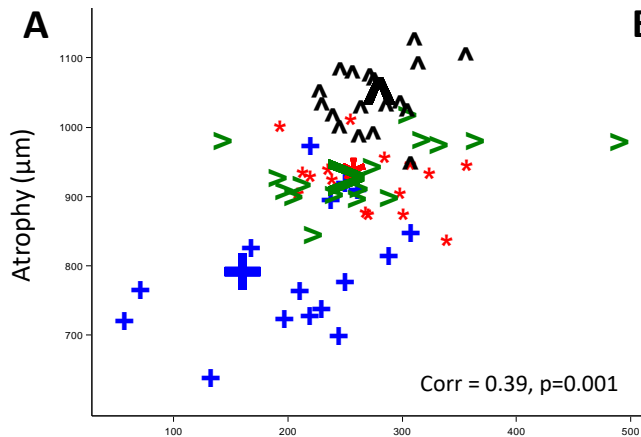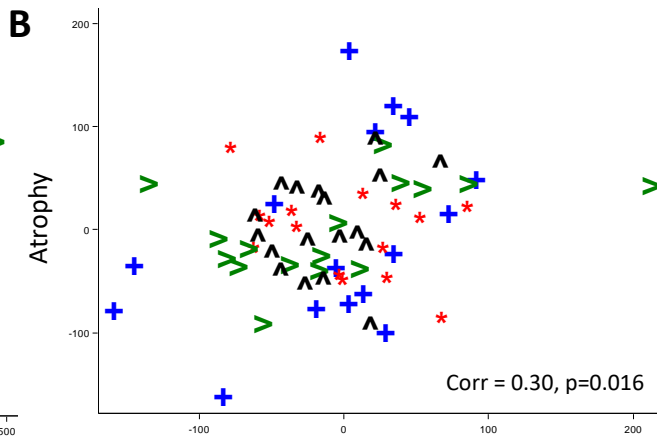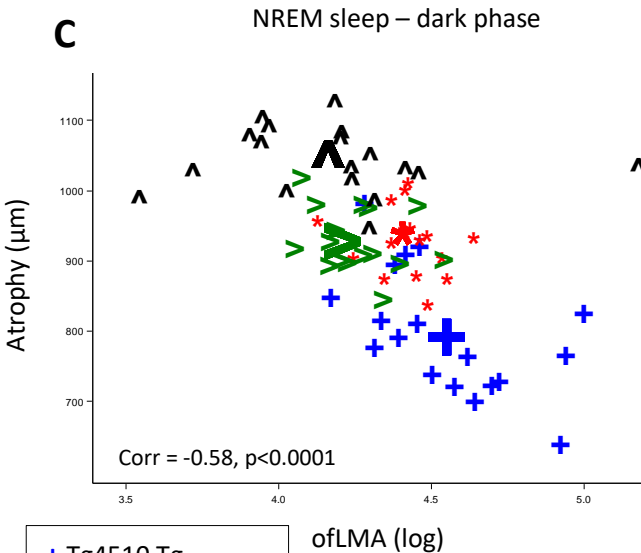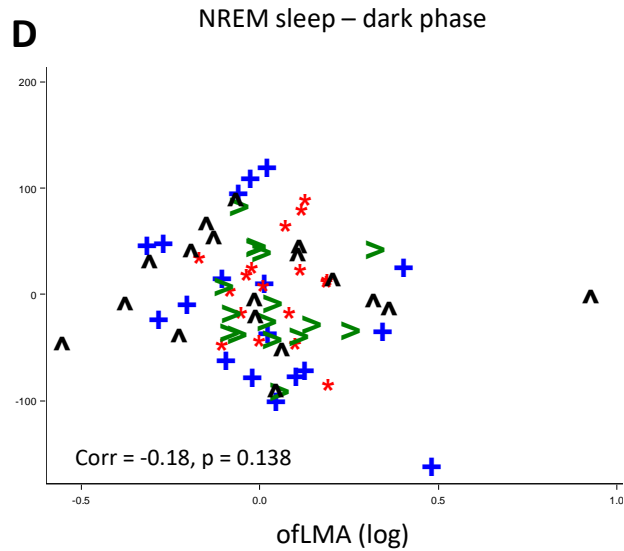

+ Tg4510 Tg  
\* Tg4510 Tg + DOX  
> Tg4510 tTA  
^ Tg4510 WT

Supplement: Supplementary file 2 — Additional file 2: Supplementary figure 2. correlation of NREM sleep or ofLMA vs atrophy. Correlation of atrophy vs NREM sleep; actual values (A) and residuals (B). Correlation of atrophy vs log distance in open field LMA; actual values (C) and residuals (D). Small symbols represent individual subjects. In the correlations with the actual values, the large symbols represent the average of the treatment group. Spearman’s correlation calculations in Table 1. [file 13195_2020_651_MOESM2_ESM.pdf]
